# Supplementary material for: Exploiting limited valence patchy particles to understand autocatalytic kinetics
Source: Nat Commun. 2018 Jul 6;9:2647. doi: 10.1038/s41467-018-04977-0 (PMC6035234; doi:10.1038/s41467-018-04977-0)
Supplement: Supplementary file 1 — Supplementary Information [file 41467_2018_4977_MOESM1_ESM.pdf]

Exploiting limited valence ‘patchy’ particles to understand autocatalytic kinetics

Corezzi et al.

Supplementary Notes

Supplementary Note 1: Expression of the autocatalytic chemical rate constant

In this Note we derive an expression for the chemical rate constant of a generic autocatalytic irreversible reaction that occurs step-wise in a binary mixture of mutually reactive monomers and which, in general, proceeds via three different reaction pathways: 1) a slower noncatalytic pathway, 2) a faster pathway catalyzed by a small amount of catalyst initially present, and 3) an autocatalytic pathway, i.e. catalyzed by a product of the same reaction. The chemical rate constant can be written as the sum of an autocatalytic (k_{auto}), a catalytic (k_{cat}), and a noncatalytic (k_{non}) contribution, i.e. $k_{chem} = \mathcal{P}_a k_{auto} + \mathcal{P}_c k_{cat} + [1 - \mathcal{P}_a - \mathcal{P}_c] k_{non}$, where $\mathcal{P}_a$ and $\mathcal{P}_c$ are the probabilities that the bonding act occurs via, respectively, the autocatalytic and the catalytic pathway of reaction. Let ΔG_n be the free energy barrier for bond formation not mediated by a catalyst, and $\Delta G_a < \Delta G_n$ and $\Delta G_c < \Delta G_n$ be the free energy barrier in the presence of the autocatalyst and the initial catalyst, respectively, i.e.

$$k_{non} = \tau_n^{-1} e^{-\beta \Delta G_n}$$

$$k_{auto} = \tau_a^{-1} e^{-\beta \Delta G_a}$$

$$k_{cat} = \tau_c^{-1} e^{-\beta \Delta G_c}$$

with τ_n, τ_a, τ_c characteristic times for the individual chemical processes. The probability $\mathcal{P}_c$ of finding the initial catalyst within an active distance R_c from the reactive sites, can be calculated assuming that the spatial distribution of catalyst is uniform. Since the probability $\mathcal{P}(N, R_c)$ of finding N catalyst units within a sphere of radius R_c will be a binomial distribution (which is the probability of finding N units within a sphere of radius R_c after a number of insertion trials N_c equal to the total number of catalyst units present in the system) one has:

$$\mathcal{P}(N, R_c) = \binom{N_c}{N} \rho^N (1 - \rho)^{N_c - N}$$

where $\rho = 4\pi R_c^3 / 3V$ is the probability of finding a catalyst within a sphere of radius R_c, inserting it randomly within a box of volume V. The probability of finding at least one catalyst unit within a distance R_c, will be:

$$\mathcal{P}_c = \mathcal{P}(N \geq 1, R_c) = 1 - \mathcal{P}(0, R_c) = 1 - (1 - \rho)^{N_c}$$

Assuming $\rho \ll 1$, i.e. the catalyst agent must be very close to the reacting sites to catalyze their reaction, one has:

$$\mathcal{P}_c \approx \rho N_c$$

In a similar way, assuming a uniform spatial distribution of the autocatalyst, the probability $\mathcal{P}_a$ of finding the autoproduced catalyst within an active distance R_c can be calculated as:

$$\mathcal{P}_a \approx \rho N_b = \rho N_A f_A p$$

where $N_b = N_A f_A p$ is the total number of bonds which are formed in a stoichiometric mixture consisting of N_A monomers with functionality f_A and N_B monomers with functionality f_B. With these expressions, the chemical rate constant for the process is:

$$\begin{aligned} k_{chem} &= \rho N_c \tau_c^{-1} e^{-\beta \Delta G_c} + (1 - \rho N_c) \tau_n^{-1} e^{-\beta \Delta G_n} \\ &+ \rho N_A f_A (\tau_a^{-1} e^{-\beta \Delta G_a} - \tau_n^{-1} e^{-\beta \Delta G_n}) p \end{aligned}$$

which can be written:

$$k_{chem} = k_1 + k_2 p = k_1(1 + \xi p)$$

by setting

$$k_1 = \rho N_c \tau_c^{-1} e^{-\beta \Delta G_c} + (1 - \rho N_c)\tau_n^{-1} e^{-\beta \Delta G_n}$$

$$k_2 = \rho N_A f_A (\tau_a^{-1} e^{-\beta \Delta G_a} - \tau_n^{-1} e^{-\beta \Delta G_n}) p$$

and

$$\xi = \frac{k_2}{k_1}$$

In the following, we make explicit these quantities in the two scenarios to which we refer in the main article.

Scenario 1. *The reaction between A and B species naturally starts via a noncatalyzed pathway, with no added catalyst.* In this case, $N_c = 0$, and then:

$$k_1 = k_{non} \quad , \quad k_2 = \rho N_A f_A (k_{auto} - k_{non})$$

$$\xi = \rho N_A f_A \left[\frac{\tau_n}{\tau_a} e^{\beta(\Delta G_n - \Delta G_a)} - 1 \right]$$

Accordingly, the chemical rate constant can be written as $k_{chem} = \tau_0^{-1} e^{-\beta \Delta G}$ with

$$\tau_0 = \tau_n \quad , \quad \Delta G = \Delta G_n$$

Scenario 2. *The reaction is too slow to occur naturally, and is initiated by a small amount of catalyst that remains unchanged throughout the reaction.* Since a catalyst is needed for the reaction to occur, $k_{non} = 0$, and then:

$$k_1 = \rho N_c k_{cat} \quad , \quad k_2 = \rho N_A f_A k_{auto}$$

$$\xi = \frac{N_A f_A \tau_c}{N_c \tau_a} e^{\beta(\Delta G_c - \Delta G_a)}$$

Accordingly, the chemical rate constant can be written as $k_{chem} = \tau_0^{-1} e^{-\beta \Delta G}$ with

$$\tau_0 = \tau_c / \rho N_c \quad , \quad \Delta G = \Delta G_c$$

Note that, in both scenarios, at any given T the value of ξ depends on the reduction in energy barrier for bond formation due to the action of the autocatalytic agent.

Supplementary Note 2: Smoluchowski diffusion equation for a growing sink with barrier

Building on the modeling of irreversible diffusion-limited cluster aggregation proposed in ref. [1], we assume to have one spherically symmetric cluster of mass $M(t)$ and radius $R(t)$ at the origin and we study the time evolution of the mass concentration $c(r, t)$ at distance $r > 2R(t)$ from the origin.
Under the assumption that it changes only via cluster diffusion, $c(r, t)$ has to obey the 3D Smoluchowski diffusion equation, that under an isotropic external potential $V_{ext}(r)$ can be written in spherical coordinates as follows:

$$\frac{\partial c(r,t)}{\partial t} = \nabla \cdot \left\{ D(t) e^{-\beta V_{ext}(r)} \nabla \left[e^{\beta V_{ext}(r)} c(r,t) \right] \right\} \tag{1}$$

where D is the diffusion coefficient and $\beta = 1/k_BT$. We observe that since both c and V_{ext} are assumed to depend only on the radial distance r, one has that $\nabla\cdot = \frac{1}{r^2}\frac{\partial}{\partial r}r^2$ and $\nabla = \frac{\partial}{\partial r}$, so that eq. (1) reduces to a one dimensional equation. Following ref. [?], we further assume that both D and V_{ext} depend on time. The external potential V_{ext}, shown in Supplementary Fig. 4a, is defined as

$$V_{ext}(r,t) = \frac{V_0}{2}\left\{1 - \tanh\left[\frac{r - L_r(t) - \Delta r}{\delta}\right]\right\}$$

where Δr is the fixed width of the barrier, δ sets the barrier steepness, and $L_r(t) = 2R(t)$. As for the diffusion coefficient $D(t)$, it is assumed to depend on the mass $M(t)$ as follows:

$$D(t) = 2D_0/M(t)^\gamma$$

where the exponent γ takes into account the effects of cluster geometry. Since the mass $M(t)$ of the cluster at the origin obeys mass conservation, the evolution of $M(t)$ (i.e. the mass growth) depends on the mass flux at the moving boundary $L_r(t)$. It is therefore governed by the equation

$$\frac{dM}{dt} = 16\pi R(t)^2 D\left[\frac{\partial c(r,t)}{\partial r} + \frac{\partial V_{ext}}{\partial r}c(r,t)\right]\Bigg|_{r=L_r(t)} \quad (2)$$

where the radius $R(t)$ and the mass $M(t)$ of the average cluster are related via $[R(t)/R(0)]^{D_f} = M(t)/M(0)$, with D_f the fractal dimension of the cluster. Note that in three dimensions in the latter equation $D_f = 3$ for compact clusters, while $D_f < 3$ for fractal clusters. Since $c(r,t)$ is, by definition, the product of the number density $n(r,t)$ of clusters (i.e., the number of clusters per unit volume) times the average cluster mass $M(t)$, we can express Eqs. (1) and (2) in terms of $n(r,t)$, thus obtaining

$$\begin{aligned}\frac{\partial n}{\partial t} &= \nabla\cdot\left\{D(t)e^{-\beta V_{ext}}\nabla\left[e^{\beta V_{ext}}n(r,t)\right]\right\} - \frac{n(r,t)}{M(t)}\frac{dM}{dt} \\ \frac{dM}{dt} &= 16\pi R(t)^2 DM\left[\frac{\partial n}{\partial r} + \frac{\partial V_{ext}}{\partial r}n(r,t)\right]\Bigg|_{r=L_r(t)} \quad (3)\end{aligned}$$

Eqs. (3) are integrated numerically with the boundary conditions $n(L_r,t) = 0$ (i.e. absorption boundary condition, since cluster aggregation is irreversible), $n(r,0) = n_0$, $n(\infty,t) = n_0$ (i.e. constant density boundary condition at infinity) and $M(0) = 1$.

We use reduced units, i.e., we measure energy in units of k_BT, length in units of Δ_r and diffusion coefficients in units of D, and solve Eqs. (3) for several values of V_0, with $\gamma = 1.75$, $D_f = 1.9$, $n_0 = 0.001$, $D_0 = 0.01$, $R(0) = 1.5$ and $\delta = 0.05$. From these numerical calculations we obtain the aggregation rate k_D, i.e.

$$k_D(M) = 4\pi L_r(t)^2 D\left[\frac{\partial n(r,t)}{\partial r} + \frac{\partial V_{ext}}{\partial r}n(r,t)\right]\Bigg|_{r=L_r(t)}$$

In Supplementary Fig. 4b this quantity is plotted against M, while in Supplementary Fig. 4c we plot the quantity

$$\zeta = e^{\beta V_0}k_D(M)/k_D^0 \quad (4)$$

with k_D^0 the aggregation rate for $V_0 = 0$. From Supplementary Fig. 4c we can argue that k_D does not scale as $e^{-\beta V_0}$ but instead the barrier can be accounted for through the M dependent factor $\zeta(M,V_0)$, always greater than 1. From eq. (4) one has

$$k_D(M) = e^{-\beta V_0}k_D^0\zeta(M,V_0) \quad (5)$$

Note that in limited valence systems, i.e. with a finite number of bonding sites, the average cluster mass can be expressed in terms of the fraction of bonds p, thus the factor ζ in eq. (5) is a function of p, i.e. $\zeta = \zeta(p,V_0)$.

Supplementary References

[1] Sciortino, F., Belloni, A. & Tartaglia, P. Irreversible diffusion-limited cluster aggregation: The behavior of the scattered intensity. *Phys. Rev. E* **52,** 4068–4079 (1995).

Supplementary Figures

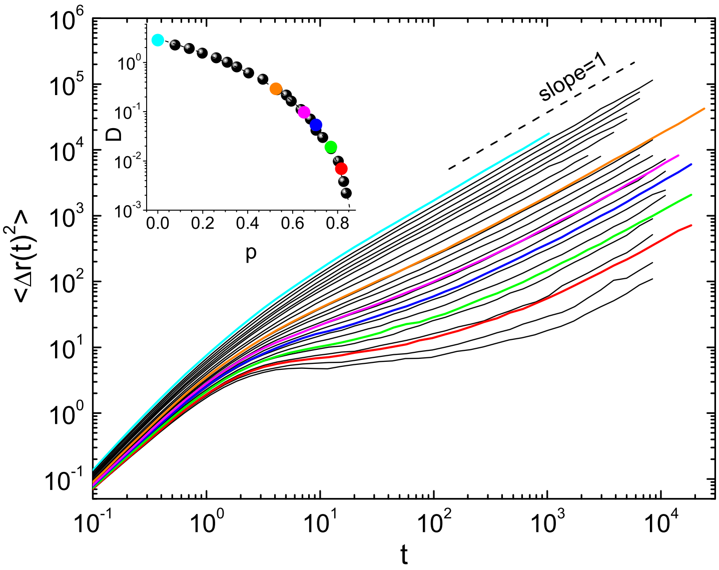

Supplementary Figure 1: Diffusivity of the system. Mean-squared displacements for configurations at different fractions of formed bonds (increasing from top to bottom) in the system without barrier (black lines) and the system with barrier $\beta\Delta U = 4$ (colored lines). A slope equal to 1 is expected when particles are diffusive. The inset shows, in the same colors, the corresponding diffusion coefficient D as a function of p.

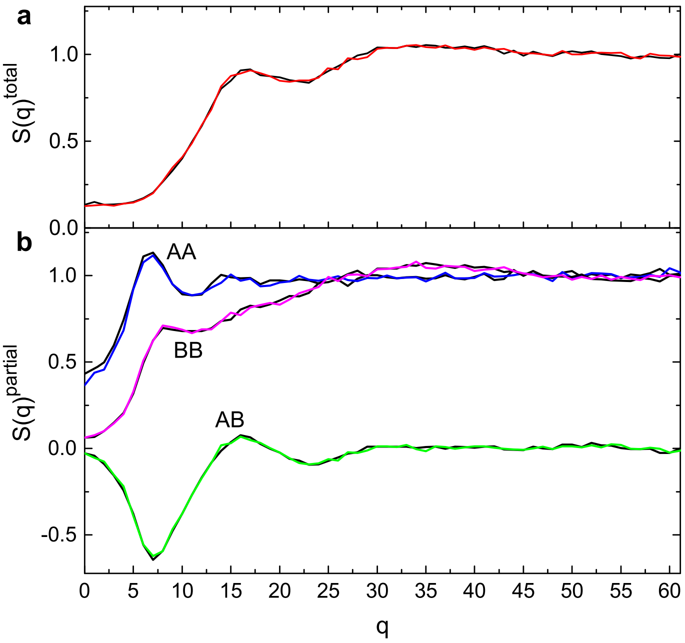

Supplementary Figure 2: Structure of the system. (a) Static structure factor $S(q)$ and (b) its partial contributions from A and B particles, when the fraction of formed bonds is $p = 0.5$. Black lines refer to the system without barrier, and colored lines to the system with barrier $\beta\Delta U = 4$. The comparison provides evidence that the structure does not change in the presence of an activation barrier.

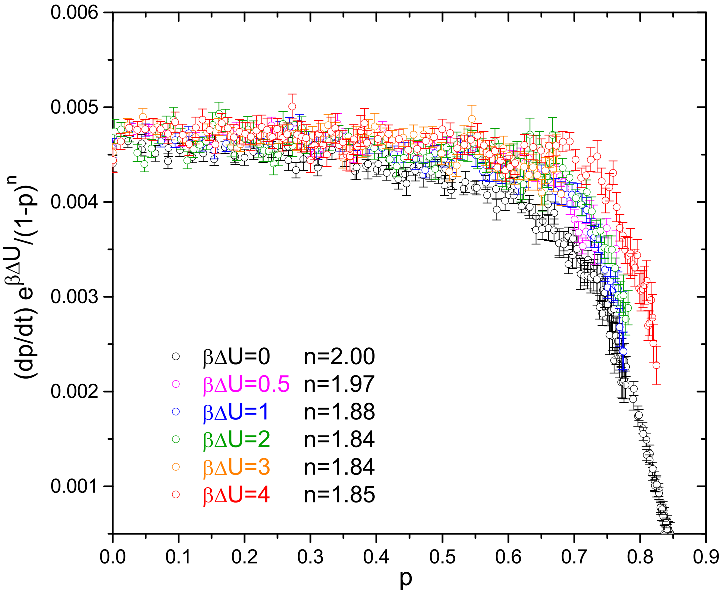

Supplementary Figure 3: Scaling of reaction rates. Reaction rate dp/dt for different values $\beta\Delta U$ of energy barrier, scaled by $(1-p)^n e^{-\beta\Delta U}$ with n a barrier dependent exponent (as indicated).

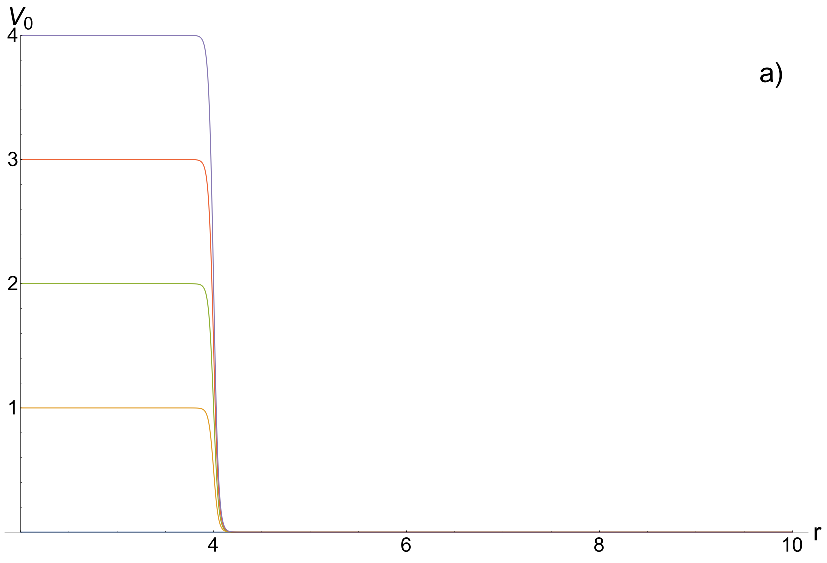

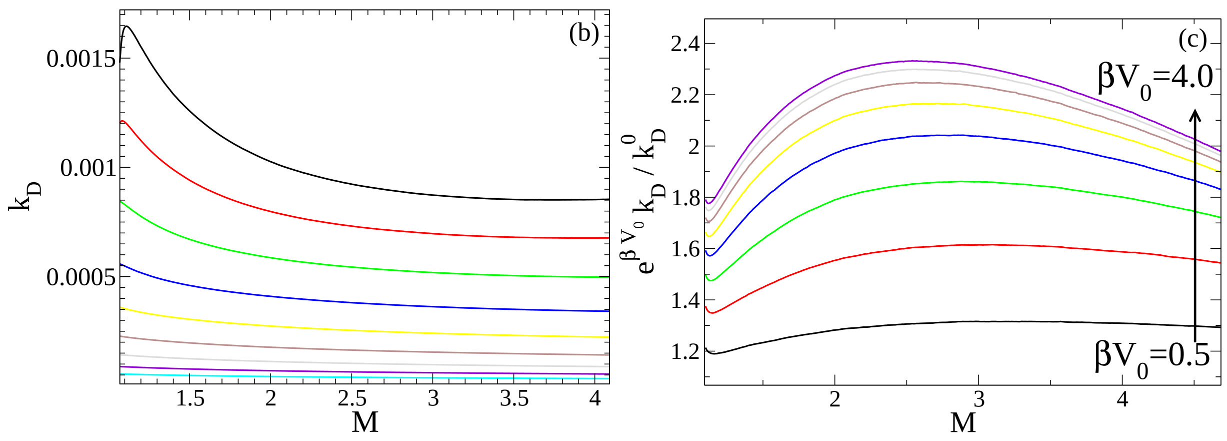

Supplementary Figure 4: Numerical solution of the Smoluchowski diffusion equation. (a) External potential used. (b) Aggregation rate k_D and (c) its ratio to $k_D^0 e^{-\beta V_0}$, against the average cluster size M for several values of βV_0.
